# Tumor-associated macrophages and individual chemo-susceptibility are influenced by iron chelation in human slice cultures of gastric cancer

**DOI:** 10.18632/oncotarget.27089

**Published:** 2019-07-30

**Authors:** Sebastian Prill, Jakob Rebstock, Anja Tennemann, Justus Körfer, Rasmus Sönnichsen, René Thieme, Ines Gockel, Orestis Lyros, Astrid Monecke, Christian Wittekind, Arved Weimann, Kerstin Grosser, Volker Wiechmann, Christoph Kubick, Ingo Bechmann, Florian Lordick, Sonja Kallendrusch

**Affiliations:** ^1^ Institute of Anatomy, University Medicine Leipzig, Leipzig, Germany; ^2^ University Cancer Center Leipzig (UCCL), University Medicine Leipzig, Leipzig, Germany; ^3^ Department of Visceral, Transplant, Thoracic and Vascular Surgery, University Medicine Leipzig, Leipzig, Germany; ^4^ Institute of Pathology, University Medicine Leipzig, Leipzig, Germany; ^5^ Department of General and Visceral Surgery, Klinikum St. Georg, Leipzig, Germany; ^6^ Department of Pathology, Klinikum St. Georg, Leipzig, Germany; ^7^ Department of Hematology and Medical Oncology, University Medicine Leipzig, Leipzig, Germany

**Keywords:** tumor-associated macrophages, tumor slice cultures, deferoxamine, gastric cancer, iron

## Abstract

**Purpose:** Presence of tumor-associated macrophages (TAM) and high levels of ferritin and lipocalin 2 (Lcn2) in the tumor microenvironment are associated with poor prognosis in many types of cancer. Here we investigate whether iron deprivation influences TAM phenotype and chemotherapy resistance in tumor slice cultures (TSC) of gastric cancer.

**Results:** TAM remained morphologically and functionally stable for four DIV. DFO treatment for 72 h decreased ferritin expression in TAM and in the tumor stroma but did not alter Lcn2 expression. TAM phenotype was altered after 72 h of cisplatin or DFO treatment compared with control conditions. Single DFO treatment and combined treatment with cytotoxic drugs significantly increased tumor cell apoptosis in TSC of gastric cancer.

**Methods:** TSC were manufactured by cutting tissue of gastric cancer resection specimens in 350 μm thick slices and cultivating them under standard conditions on a filter membrane, at an air-liquid interface. After 24 h *ex vivo*, TSC were treated with irinotecan (100 nM) or cisplatin (10 μM) alone and in combination with deferoxamine (DFO; 10 μM, 100 μM), respectively, for 72 h. After four days *in vitro* (DIV) the TSC were fixated with paraformaldehyde, paraffin embedded and analyzed by immunohistochemistry for apoptosis (cPARP), proliferation (Ki67), TAM (CD68, CD163), ferritin, and Lcn2 expression.

**Conclusions:** TAM are well preserved and can be studied in TSC of gastric cancer. Iron deprivation significantly increased tumor cell apoptosis.

## INTRODUCTION

Gastric cancer is the fifth most common tumor disease and the third most common cause of cancer-related death worldwide [1]. Still, response to cytotoxic treatment, which is the standard of care in the adjuvant and palliative setting, is unpredictable [2]. The tumor microenvironment plays an important role in tumor progression and treatment resistance [3–5]. Immune cells and fibroblasts in the TME promote tumor cells by wound healing processes [5]. One important cell population in the tumor microenvironment are tumor-associated macrophages [6, 7]. It has been shown that the number of TAM, a specialized population of macrophages, in gastric cancer is negatively correlated with patients' overall survival [8–10]. Macrophages have shown big differences in their phenotype and function [11]. Macrophages display a continuum, ranging from classically-activated-, inflammatory-, M1 phenotype to alternative-activated-, anti-inflammatory-, or M2 phenotype [11]. In comparison to physiologic inflammatory, M1 phenotype tumor supportive TAM phenotype is rather assigned to the M2 phenotype. Apoptotic tumor cells are able to trigger TAM to an iron-release phenotype (mainly M2), as macrophages are main regulators of iron within tissue and the overall systemic homeostasis [6, 7, 12]. These TAM secrete iron, lipocalin 2 and ferritin into the tumor stroma, which increases tumor cell proliferation and metastasis [6, 7, 13, 14]. Studies in cell lines and primed monocytes or macrophages, have shown that iron chelators might be used as anti-cancer agents and are able to change the macrophage phenotype towards iron sequestration [12, 15–19]. However, experiments with cancer cell lines and primed monocytes do not appropriately mirror the major in-vivo heterogeneity of tumor and stromal cells. Long-established cell lines differ significantly from the primary tumor, and the process of cultivation selects and homogenizes these cells [20–22]. Other clinically relevant models are needed for the investigation of interactions between tumor cells and TAM in a model that reflects more the spectrum of inter- and intratumoral heterogeneity. Here we explored the interaction between deferoxamine, cancer cells, apoptosis and TAM in the human slice culture model of gastric cancer.

## RESULTS

**Figure 1:**
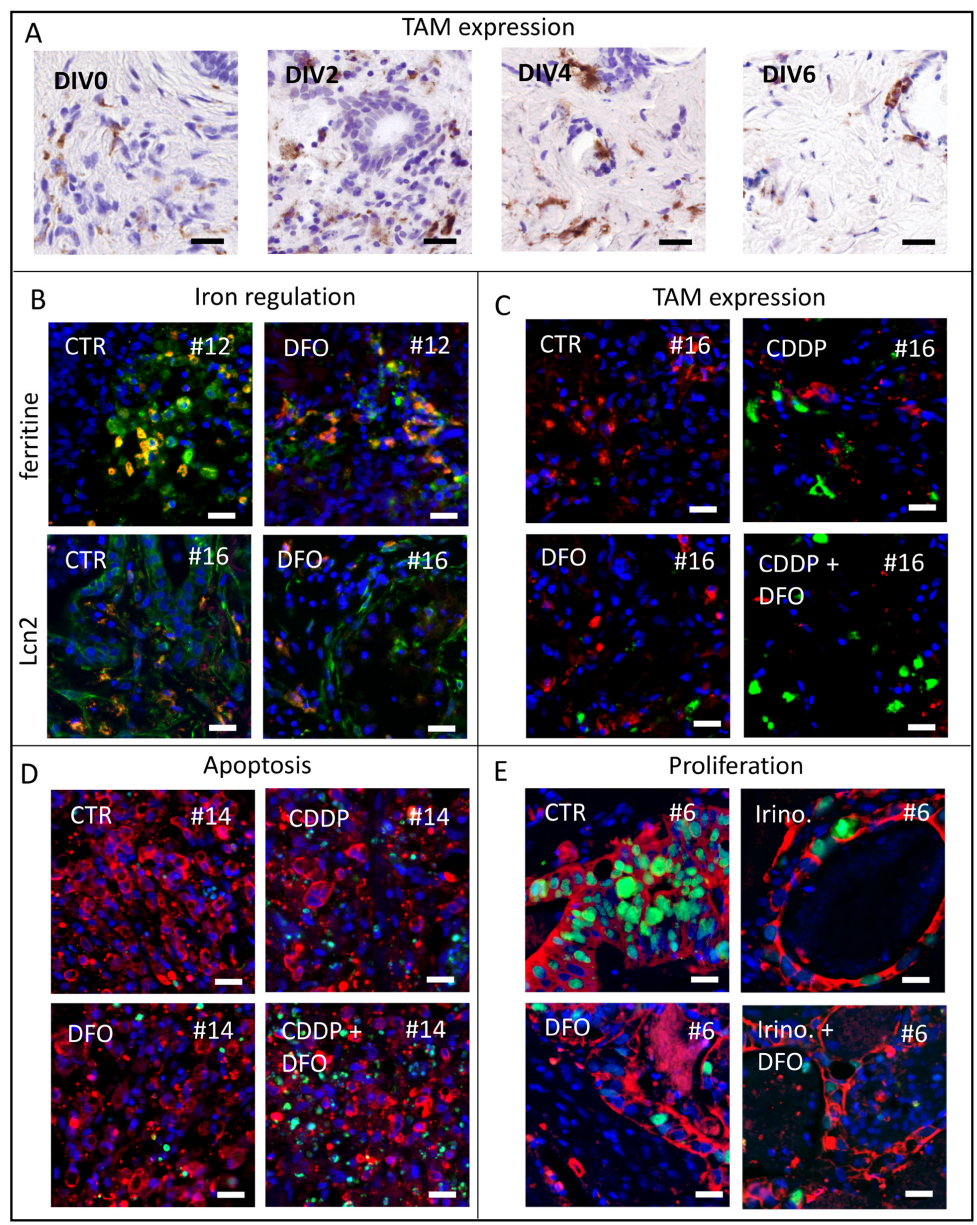
Representative pictures of analyzed human tumor slice cultures. (**A**) To investigate macrophage survival we use CD68 antibody staining for TAM *in vitro* after 0, 2, 4, 6 DIV of gastric cancer specimens. (**B**) Representative images of ferritin expression (green) and co-located TAM (CD68, red) after 4 DIV in control and DFO treated conditions of specimen #12 and representative images of Lcn2 expression (green) and co-located TAM (CD68, red) after four DIV in control and DFO treated conditions of specimen #16 (Hoechst, blue). (**C**) Representative pictures of macrophage phenotypes (CD68, red; CD163, green) expression after treatment at four DIV in specimen #16 (Hoechst, blue). (**D**) Representative images of apoptotic tumor cell fraction (Hoechst, blue; AE 1/3, red; cPARP, green) of specimen #14. (**E**) Representative images of proliferating tumor cell fraction (Hoechst, blue; AE 1/3, red; Ki67, green) of specimen #6. (Bars 20 μm).

Representative pictures of TAM in TSC of gastric cancer (Figure 1A), Lcn2 expression (Figure 1B), ferritin expression (Figure 1B), TAM expression (Figure 1C), apoptosis (Figure 1D) and proliferation analysis (Figure 1E) are shown in Figure 1.

### TAM remain morphologically and functionally stable until day four *in vitro*

**Figure 2:**
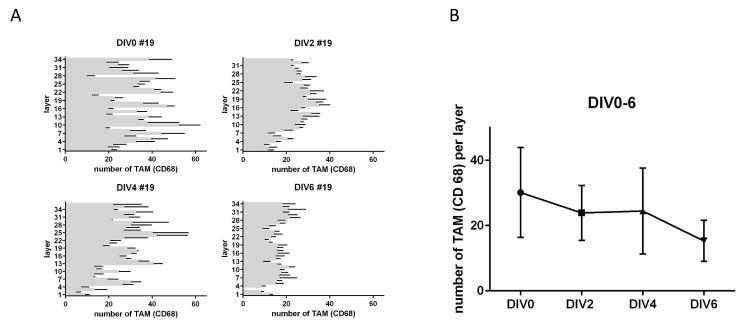
TAM in tumor slice cultures. (**A**) Number of CD68 positive cells per layer in tumor slice cultures from top to bottom after DIV2-6. (**B**) Total amount of CD68 after 0, 2, 4, 6 DIV (*n* = 1). (Representative pictures are shown in Figure 1A).

In order to test for changes in TAM (CD68) expression after treatment, we first investigated the preservation of macrophages in their morphology and number in human gastric cancer slice cultures. In addition, we wanted to know whether TAM accumulate in certain areas in TSC under culture conditions. This is fundamental for determining the survival and functional state of the macrophage fraction, as well as for analyzing the adequate paraffin sections to standardize macrophage analysis. Morphologically we identified intact cell bodies of CD68-positive TAM with co-located nuclei until day four *in vitro* (Figure 1A). After six DIV we saw partially interrupted cell bodies of TAM (CD68) with no or small nuclei (Figure 1A). The number of TAM did not show any significant change throughout TSCs up to six DIV (Figure 2A). However, the mean amount of TAM shows a decreasing trend after DIV4 (Figure 1B). Confocal live microscopy identified vital and migratory TAM (IB4) until DIV4 (Supplementary Figure 1).

### DFO increases iron in culture medium

**Figure 3:**
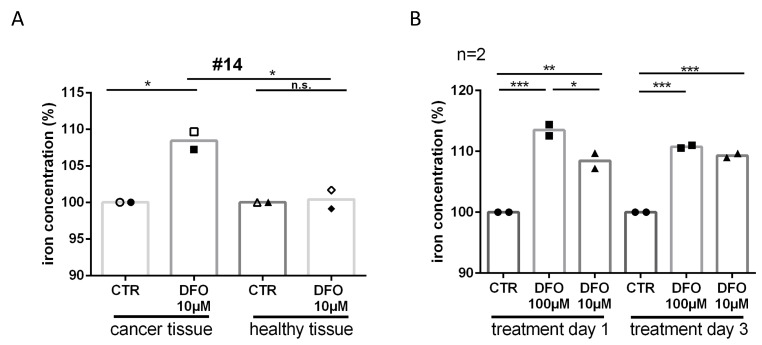
Iron deprivation after DFO treatment. (**A**) To check if DFO treatment removes iron from the tissue we had the medium analyzed by inductively coupled plasma mass spectrometry. Relative iron concentration is shown after single day donation in the medium of slice cultures in tumor and adjacent healthy tissue after 24 h (filled icons) and 72 h (closed icons) DFO (10 μM) treatment from specimen #14. (**B**) Relative iron concentration after single day donation in the medium of tumor slice cultures after 24 h and 72 h DFO (100 μM, 10 μM) treatment.

To explore whether iron was being removed from the tissue due to the DFO treatment, we examined the iron concentration of the slice culture media. The content of iron was significantly increased after DFO 100 μM (24 h treatment, Δ13.5%, *P ≤* 0.001; 72 h treatment, Δ11%, *P ≤* 0.001) and DFO 10 μM treatment (24 h treatment, D8.5%, *P ≤* 0.01; 72 h treatment, D9.5%, *P ≤* 0.001) in culture medium compared with control media (Figure 3B). In addition, we could show that the iron concentration in culture media of tumor tissue was significantly higher than in media of adjacent healthy tissue (D8%, *P ≤* 0.05) after DFO 10 μM treatment (Figure 3A). The iron concentration in culture media of adjacent healthy tissue was not increased after treatment with DFO 10 μM compared with controls (Δ0.5%, n.s.). In addition, 10 μM DFO treatment increased apoptosis of tumor cells (Δ2%, n.s.) but not the apoptosis in adjacent healthy tissue (Δ0.5%, n.s.) compared with the respective controls (Supplementary Figure 2).

### Lcn2 and ferritin expression

**Figure 4:**
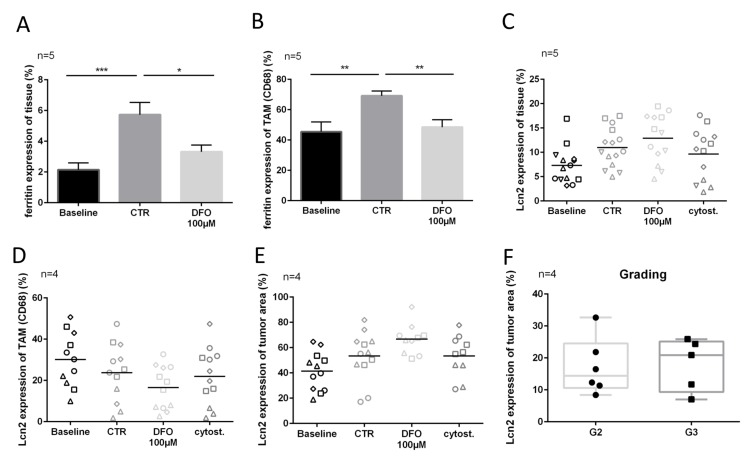
Lcn2 and ferritin expression after chelation therapy. (**A**) Ferritin expression in overall tissue (measured in entire Hoechst area). (**B**) Ferritin expression in TAM (colocalized with CD68). (**C**–**E**) Lcn2 expression of overall tissue (measured in entire Hoechst area), TAM (colocalized with CD68) and tumor cells (colocalized with AE 1/3 area) after four DIV. (**F**) Lcn2 expression of tumor cells on preparation day (DIV0) in order to grading (G2, G3). (Representative pictures are shown in Figure 1).

Ferritin expression of tissue treated with 100 μM DFO was significantly lower (∆2.5%, *P ≤* 0.05) than in the control tissue (Figure 4A). Similar effects could be observed in CD68 expressing TAM (ferritin expression: CTR vs. DFO 100 μM, ∆20.5%, *P ≤* 0.01) (Figure 4B). The expression of Lcn2 showed no changes between treated conditions and controls, nor in tumor cells nor in TAM (Figure 4C–4E). However, the Lcn2 expression of tumor cells was very individual, and without association with the proliferating tumor cell fraction or apoptotic tumor cell fraction (Figure 4). There was a trend towards higher median Lcn2 expression in the here investigated G3 tumors as compared with the median of G2 tumors (Figure 4F).

### Chemotherapy induced apoptosis and iron chelator treatment influence TAM-phenotype

**Figure 5:**
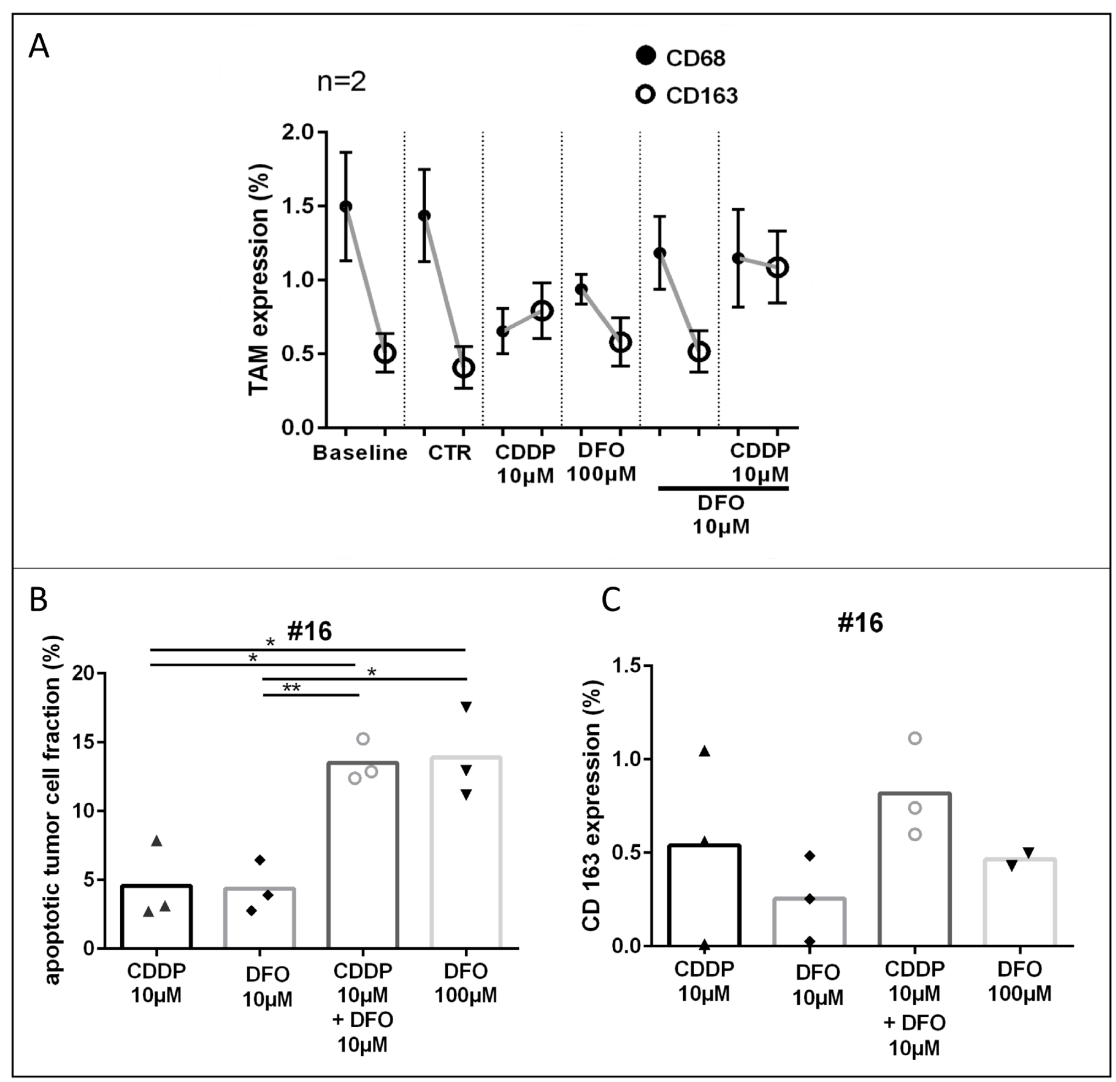
TAM-phenotype expression after cisplatin and DFO treatment. (**A**) TAM (CD68; CD163) expression in overall tumor tissue after treatment. (**B**, **C**) Apoptotic tumor cell fraction and CD163 expression of specimen #16. (Representative pictures are shown in Figure 1).

Three cases of gastric cancer were examined for CD68 and CD163 expression in the tumor stroma. Graphs are shown in Figure 5A (two chemo-naive specimens; #14, #16) and in Supplementary Figure 3 (one neoadjuvant treated specimen; #12). In two pooled chemo-naive cases no change of CD68 and CD163 expression was observed in the control conditions (DIV4, CD68: Δ0.1%; CD163: Δ0.1%) compared to the baseline tissue (DIV0, Figure 3B; #14, #16). In the chemo-naive specimens, treatment with cisplatin led to decreased expression of CD68 and an increase of the CD163 expression compared with controls, these changes were, however, not significant (CD68: Δ0.8%; CD163: Δ0.4%). Compared to controls, DFO treated conditions (100 μM and 10 μM) depicted lower CD68 expression and similar CD163 expression (DFO 100 μM CD68: 0.3%, CD163 0.1%; DF0 10 μM 0.3%, CD163: 0.1%). DFO treatment resulted in enhanced CD68 expression and lower CD163 expressions (DFO 100 μM: CD68: Δ0.3%, CD163: Δ0.2%; DFO 10 μM: CD68: Δ0.5%, CD163: Δ0.3%) compared with cytostatic cisplatin treatment. The combined treatment of DFO and cisplatin led to lower CD68 expression and increased CD163 expression compared with the control conditions (CD68: Δ0.3%; CD163: Δ0.7%). One neoadjuvant treated specimen (Supplementary Figure 3; #12) depicted similar changes in TAM phenotype expression as described above could be observed, shown in Supplementary Figure 3 (irinotecan 100nM: CD68: 0.4%; CD163: 0.1%; DFO 100 μM: CD68: 0.4%; CD163: 0.5%; DFO 100 μM + irinotecan 100nM: CD68: 0.3%; CD163: 0.2%; all conditions compared with controls).

CD163 expression is increased by apoptosis. In order to consider the induction of CD163 expression by apoptosis we compared the conditions of equal apoptotic tumor cell fractions with CD163 expression. In case #16 similar apoptotic rates were observed in the CDDP 10 μM and DFO 10 μM treated conditions (∆0.2%) or in the DFO 100 μM + CDDP 10 μM and DFO 100 μM treated conditions (∆0.4%, Figure 5B). CD163 was in both CDDP treated conditions higher expressed than in the low or high DFO treated conditions (DFO 10 μM, ∆0.3%; DFO 100 μM, ∆0.4%) compared with conditions with similar apoptotic tumor cell fraction and lower DFO concentration (CDDP 10 μM; DFO 10 μM + CDDP 10 μM; Figure 5C).

### Tumor slice cultures respond to chelator treatment

**Figure 6:**
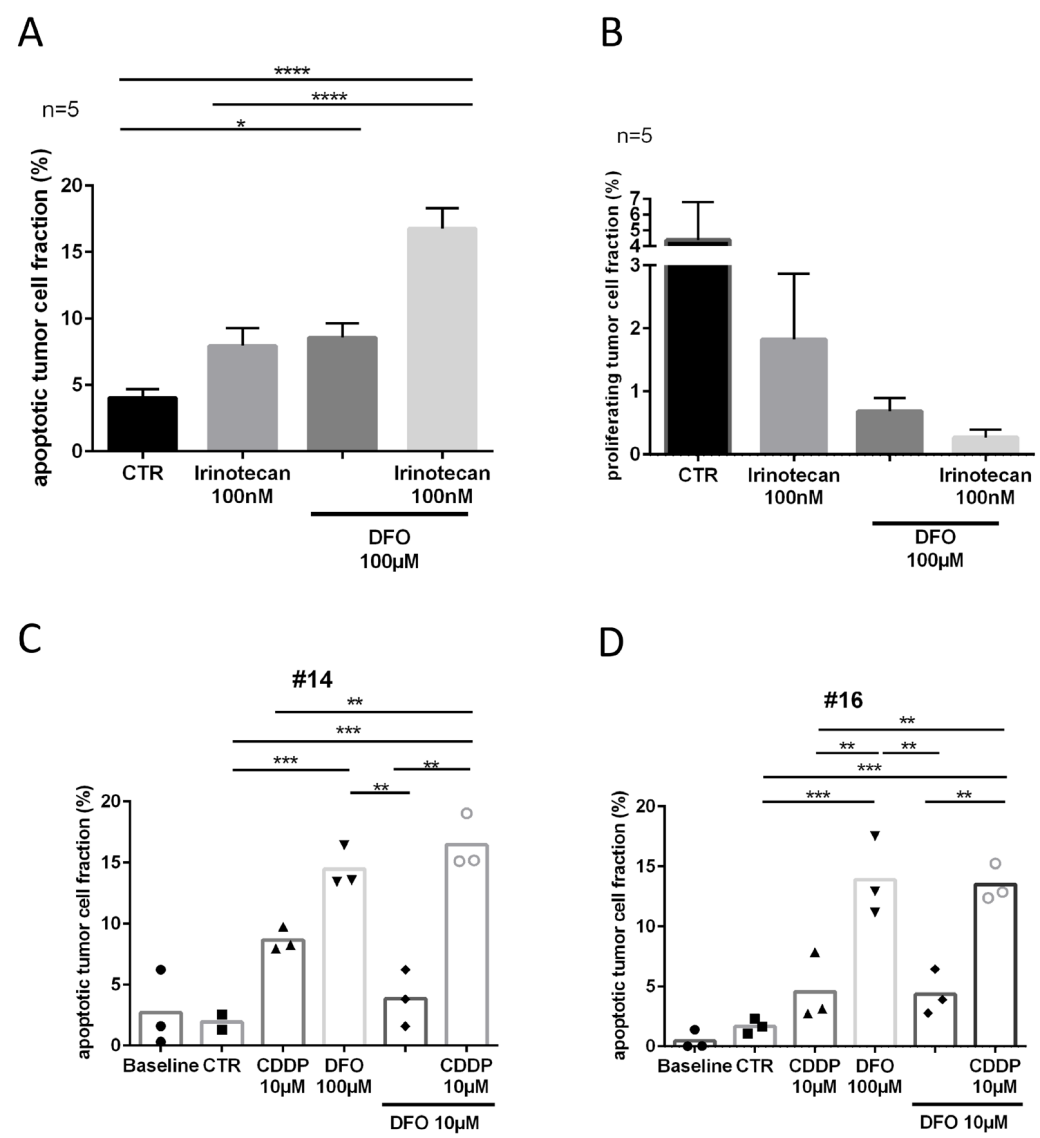
Tumor response to DFO and cytostatic treatment. (**A**) Apoptotic tumor cell fraction and (**B**) proliferating tumor cell fraction after four DIV. (**C**, **D**) Apoptotic tumor cell fractions of chemo-naive patients, specimen #14 and specimen #16 after four DIV. (Representative pictures are shown in Figure 1).

Proliferation analysis presented very heterogeneous inter- and intratumoral results in slice cultures of gastric cancer. Here only trends indicated decreased proliferating tumor cell fractions in the treated conditions compared to the control conditions (irinotecan, Δ2.5%; DFO, Δ3.5%; irinotecan and DFO, D4%; Figure 6B). However, treatment replicates (DFO 100 μM) of slice culture conditions showed comparable means of apoptotic tumor cell fraction (Δ0.3%; Supplementary Figure 5).

Five samples of patients who received neoadjuvant chemotherapy (clinical data is shown in Supplementary Table 1) were treated with DFO 100 μM and showed a significant increase in apoptotic tumor cell fraction (D4.5%, *P ≤* 0.05) compared with the control conditions (Figure 6A). The combination of DFO 100 μM and irinotecan 100 nM provoked a further significant increase in apoptotic tumor cell fraction compared with irinotecan 100 nM alone (D9%, *P* ≤ 0.0001) or the control condition (Δ12.5%, *P ≤* 0.0001). Two chemo-naive specimens (#14, #16) were treated with DFO and CDDP to determine chemotherapeutic susceptibility (Figure 6C, 6D). Case 16 (#16, Δ3%, n.s.) showed less apoptotic tumor cell fraction after CDDP treatment than case 14 (#14, D6.5%, n.s.) in comparison with their respective controls. However, specimens #14 and #16 showed comparable changes in DFO 100 μM treated conditions compared with controls (#14, Δ12.5%, *P ≤* 0.001; #16, Δ12%, *P ≤* 0.001), CDDP 10 μM (#14, D6%, n.s.; #16, D9%, *P ≤* 0.01) and DFO 10 μM treated conditions (#14, Δ10.5%, *P ≤* 0.01; #16, 9.5%, *P ≤* 0.01). Conditions which were treated with the combination of CDDP 10 μM and DFO 10 μM had a significantly higher apoptotic tumor cell fraction than CDDP 10 μM (#14, D8%, *P ≤* 0.01; #16, D9%, *P ≤* 0.01) alone or single DFO 10 μM treatment (#14, Δ12.5%, *P ≤* 0.01; #16, D9%, *P ≤* 0.01). In specimen #16 trends subsequently indicated decreased proliferating tumor cell fractions in the treated conditions compared to the control conditions (CDDP 10 μM, D6%; DFO 100 μM, D8%; DFO 10 μM, D6%; CDDP 10 μM + DFO 10 μM, D8%; Supplementary Figure 4).

## DISCUSSION

Several *ex vivo* tumor slice culture models for tumor entities are already established and first correlations with clinical data exist [23–31]. We here investigated the preservation of TAM in human gastric TSC and studied their phenotype. We expected that iron chelation in TSC would alter the TAM phenotype and thereby will influence tumor cell survival in human gastric TSC.

TAM are mostly studied by converting monocytes with LPS and IFN-γ or IL-4/ IL-10 to an excessed macrophage phenotype [12, 15]. With this procedure, a low heterogeneity in macrophage phenotype is generated and the natural heterogeneity of macrophages is underestimated. To the best of our knowledge, our experiments show for the first time that human TAM remain stable in their morphology, number and activity in gastric cancer slice cultures until day four *ex vivo*. In human slice cultures, we expect a great variety of TAM phenotypes, because of the possible crosstalk between TAM, cancer cells and other stromal cells.

A physiological function of macrophages is to maintain the iron balance in human tissue. Increased iron traffic by TAM is discussed to promote tumor progression or tumor protraction. Here, we investigated TAM regulation by applying the clinically approved iron chelator DFO. First, it was demonstrated that DFO deprives iron from cancer tissue of slice cultures, but not from slice cultures of adjacent gastric cancer tissue. *In vivo*, DFO is given systemically and will not only accumulate in tumor tissue. Therefore, the influence on healthy tissue is essential for safe administration. These results are in line with the literature, where cancer cell lines demonstrate reduced viability and high iron efflux with chelator treatment whereas this could not be found in non-cancer cell lines [16, 32, 33]. Likewise clinical studies exhibited increased urinary iron excretion in cancer patients whereas [34] urinary iron excretion was not significantly increased in healthy individuals after deferoxamine or other iron chelator therapy.

To further study the iron depletion within the tissue, ferritin expression was investigated. It was shown that ferritin supplementation provoked tumor cell proliferation in breast cancer cell lines [13]. In the present study we found that ferritin expression decreased after DFO treatment in the tissue and in TAM. The same effect was seen in an esophageal-cancer-derived xenograft mice study, where the tissue expression of ferritin was reduced after three weeks of deferasirox treatment [16]. Primary macrophages then also displayed enhanced ferritin secretion after iron supplementation [13].

Another iron regulating protein respectively a siderophore binding protein, Lcn2 was shown to potently increase the intracellular labile iron pool of cancer cells, tumor proliferation and induce chemotherapy resistance. Further, it is expressed by TAM and cancer cells [6, 14, 35–38]. Another aspect of Lcn2 is its involvement in inflammation and wound healing processes, however this protein is hardly explored and it is controversial discussed [38–40]. Difficulties to clarify the role of Lcn2 might be related to its different iron-unbound apo-lipocalin-2 and iron-bound, immune suppressive holo-lipocalin-2 form, which may have opposite functions, such as infection defense (iron depletion), wound healing (iron release) or tumor progression (iron release) [39, 41]. In the present study, Lcn2 distribution did change only in individual cases. We observed non-significantly enhanced Lcn2 expression in G3 tumors compared to G2 tumors, likewise stated in literature [42]. But data are limited for Lcn2 tumor tissue expression and the observed changes in our present study might reflect culturing artefacts, as Lcn2 is also involved in inflammation and wound healing processes [38–40].

Different macrophage phenotypes influence Lcn2 expression as they play a pivotal role in the tumor microenvironment due to their diverse functions [9, 11]. The macrophage phenotype is even used as prognostic marker in ovarian cancer patients, where a high M1/ M2-macrophages ratio is related to better survival [43]. The macrophage classification is used to categorize macrophages in their current state of action. This is considerable, regarding that apoptotic tumor cells trigger macrophages towards an iron releasing phenotype [7]. After chelator treatment a switch towards a M1 phenotype was found in cell culture experiments by analyzing RNA expressions of ferroportin by Mertens *et al* [15]. Here, we observed that high DFO supplementation reduced the CD68 expression without increasing the CD163 expression (M2), despite the increase in apoptotic tumor cell fraction. Cisplatin alone however also decreased the CD68 (M1) population but marginally enhanced the CD163 (M2) population. As described in the literature, iron chelator therapy in combination with cytotoxic treatment could reduce chemotherapy resistance [16, 19, 37]. In addition, iron chelator treatment has shown interactions with macrophage conditioned medium that affects the tumor cell count. A cell culture study where the tumor cell line RCC10 was incubated with M2 conditioned medium showed a higher cell count than in the M1 conditioned group, which was lower than in the control group. Additional DFO supplementation decreased the cell count, despite M2-conditioned medium [12]. Therefore TAM might play an important role in chemotherapy resistance. Our TSC experiments demonstrate that iron chelation alone and in combination with cytotoxic drugs show anti-tumor effects in human slice cultures of gastric cancer. However, in TSC we have a broad heterogeneity of cellular phenotypes and protein expressions. Thus a direct correlation between the phenotype alteration of TAM and tumor survival could not be shown. However, some pre-clinical and clinical trials with iron chelating agents have been accomplished for exploring the potential of cancer treatment [17, 44–48]. Although not all patients responded to the iron chelation therapy, many patients had better responses and one case, classified as chemotherapy-non-responder, showed that the primary tumor together with all lung metastases completely disappeared after DFO therapy [44].

Human TSC are an ex-vivo model which may fill the gap between cell culture and clinical studies. The tissue composition, cell-cell interactions and chemo- and cytokine environment of TSC is much more like the *in vivo* situation compared to standard cell cultures. Most components of the TME co-exists with the tumor cells. However, TSC still need to be evaluated for each different cell population. The influence of neoadjuvant therapy and surgical resections is difficult to estimate. Thus, further steps are the examination of other components of the TME like cancer-associated fibroblasts, different T-cell populations as well as blood and lymph vessels. Furthermore, the stability of the tissue in culture of a longer period should be explored. In future we need more patient-derived material together with clinical studies to identify novel therapeutic possibilities in such a heterogeneous disease like gastric cancer.

In summary, our results demonstrate that TSC of gastric cancer reproduce observations from studies done in mice and cell lines and can deliver additional results concerning the tumor-stroma interaction. Further it could be shown that iron chelation alone and in combination with cytotoxic drugs show clear anti-tumor effects in human slice cultures of gastric cancer.

## MATERIALS AND METHODS

### Specimens

Patient-derived specimens were recruited from two academic hospitals in Leipzig, Germany. Seven patients with gastric and esophagogastric junction cancer were included in this study. This study was approved by the ethics committee of the Medical Faculty, University of Leipzig. All patients provided their informed written consent.

### Preparation of tumor slice cultures

Slice cultures of gastric cancer samples were prepared as previously described [30]. Briefly, after surgical resection and pathological assessment tumor specimens were cut into 350 μm slices by using a tissue chopper (McIlwain TC752; Campden Instruments, Leicestershire, England). The slices were standardized by a 3mm coring tool (kai Europe, Solingen, Germany). Three tissue slices were randomly pooled for an experimental condition. Then tumor slices were cultivated on membrane inserts (Millipore Corporation, Billerica, MA, USA) in 6-well plates. Slices were incubated under optimized conditions of 37°C and 5% CO2. Medium was changed every 24 h. Baseline tissue was fixated with 4% paraformaldehyde at the day of the preparation and cultivated tissue was fixated overnight after 4 days *in vitro*.

### Experimental setup

The tissue of one specimen was fixated after two, four and six days *in vitro* without any treatment for TAM location analysis (image stacking). Five neoadjuvant treated specimens were treated after 24 h *in vitro* with irinotecan (SN38; 100nM, Tocris, Bristol, UK), deferoxamine (100 μM, Sigma-Aldrich, St. Louis, USA) and the combination of irinotecan (100nM) and deferoxamine (100 μM). Two chemo-naive specimens were treated with cisplatin (10 μM, Neocorp, Weilheim, Germany), deferoxamine (100 μM), deferoxamine (10 μM), and the combination of cisplatin (10 μM) and deferoxamine (10 μM). Medium was prepared at the day of preparation and the treatment took place with every medium change (24 h, 48 h, 72 h). The medium from two specimens was frozen after every medium change in liquid nitrogen and were stored at –80° C.

### Staining procedure

Tumor slice cultures were fixated overnight with 4% paraformaldehyde, paraffin-embedded and processed to 7 μm sections. After HE staining, the tumor cell proportion was surveyed in the overview. Tumor cell fraction, apoptotic tumor cell fraction, proliferating tumor cell fraction, Lcn2 expression, Ferritin expression, CD68 expression and CD163 expression on TAM were analyzed after immunofluorescence staining. Paraffin sections were deparaffinized, washed with 0.3% phosphate buffered saline/ TritonX and blocked with normal goat serum for 30 minutes before adding primary antibodies. Primary antibodies were diluted in 0.5% bovine serum albumin and incubated at 4°C overnight. We used antibodies against cytokeratins (AE1/3; BioGenex, Fremont, CA, USA, mouse, 1:100), Ki67 (DCS, Hamburg, Germany, rabbit, 1:200), cPARP (abcam, Cambridge, UK rabbit, 1:100), CD68 (Dako, mouse, 1:500), CD163 (abcam, Cambridge, UK, rabbit, 1:200), Ferritin (Sigma-Aldrich, St. Louis, USA, rabbit, 1:800) and lipocalin 2 (NGAL; R&D Systems, Minneapolis, USA, rat, 1:200). After washing with 0.3% phosphate buffered saline/TritonX sections were labeled with secondary antibodies including goat-anti-rabbit 568 (Alexa Fluor, Invitrogen, Eugene, OR, USA), goat-anti-mouse 647 (Alexa Fluor, Invitrogen, Eugene, OR) and goat-anti-rat 647 (Alexa Fluor, Jackson, West Gove, USA). Hoechst 33342 (Sigma-Aldrich, St. Louis, MO, USA) was used for nuclei staining.

### Analysis

HE sections were examined by using slide scans (Pannoramic SCAN and Pannoramic Viewer, 3D Histech, Budapest, Hungary). Nine pictures (20x) per condition (three per slice culture) were taken from fluorescent sections by using an Olympus BX51 fluorescent microscope (Olympus Deutschland, Hamburg, Germany). Positive pixels from all fluorescent stainings were counted by using specific algorithms for each stain by ImageJ (modified from Sönnichsen *et al*. 2017). Total positive pixels of Hoechst 33342, AE1/3, cPARP and Ki67 were counted for cell nuclei (Hoechst 33342-positive), tumor cell fraction (Hoechst33342-, AE1/3-positive), apoptotic tumour cell fraction (Hoechst-, AE1/3-, cPARP-postive) and proliferating tumor cell fraction (Hoechst 33342-, AE1/3-, Ki67-positive). The calculation of tumor cell fraction was based on Hoechst 33342-positive nuclei with co-localization in tumor cell area per total Hoechst 33342-positive nuclei and proliferating or apoptotic tumor cell fraction was based on calculation of co-localized Ki67- or cPARP- and Hoechst 33342-positive nuclei in the tumor cell area per total Hoechst 33342-positive nuclei in the tumor cell area. Lcn2 and ferritin expression from TAM and tumor cells were calculated by counting co-localized pixels of Lcn2 or ferritin and AE1/3 or CD68 respectively and were related to the total pixels of AE1/3 and CD68. CD68 and CD163 expression were analyzed by calculating the proportion of CD68 and CD163 positive pixels per tissue area of the picture.

### Inductively coupled plasma mass spectrometry

Iron concentration from slice culture medium was analyzed by ‘MVZ Medizinisches Labor Bremen GmbH’ from Germany. For sample preparation 100μl of the slice culture medium was diluted in 2.4ml deionized water. Samples were analyzed by an Agilent 7700x inductively coupled plasma mass spectrometer equipped with collision-/reaction cell technology. For calculation of quantitative results for Fe the isotope ^54^Fe was applied. Six calibration solutions containing 0, 1, 2, 5, 10 and 20 μg/l were used for calibration.

### Confocal live microscopy

TAM migration was observed by confocal live microscopy in 5 cases. After 24 h incubation with IB4 (Isolectin GS-IB_4_ Alexa Fluor 647 conjugate, Invitrogen, Carlsbad, USA, 1:333) standardized tumor slice cultures were merged with gastric adjacent healthy tissue cultures to co-cultures. On every day *in vitro* (DIV1-4) recordings were made in one case. In other cases, TAM migration was recorded 12 to 20 h on DIV1 (representative pictures are shown in Supplementary Figure 1).

### Statistical analysis

For combination of different experiments, mean values and SEM were calculated from mean condition values. SEM was not calculated for mean condition value of single specimens, because it only reflects intratumoral heterogeneity. In TAM phenotype expression of specimen #12 we had to calculate the SEM based on the illustration of the graph. GraphPad Prism 6 (GraphPad Software, La Jolla, VA, USA) was used for performing one-way analysis of variance with Bonferroni correction. *P* < 0.05 was considered significant.

## SUPPLEMENTARY MATERIALS



## Abbreviations

AE 1/3: pan cytokeratin marker
CD: cluster of differentiation
CDDP: cisplatin - *cis*-diamminedichloridoplatinum(II)
cPARP: cleaved Poly (ADP-ribose) polymerase
CTR: control
DFO: deferoxamine
DIV: days *in vitro*
HE: hematoxylin and eosin
IB4: isolectin GS-IB_4;_
IFN-γ: interferon gamma
IL: interleukin
Ki67: MKI67, proliferation marker
LPS: lipopolysaccharide
Lcn2: lipocalin 2
LIP: labile iron pool
NGAL: neutrophil gelatinase-associated lipocalin
PFA: paraformaldehyde
RCC10: renal cell carcinoma (human)
SEM: standard error of the mean
SN38: DNA topoisomerase inhibitor, active metabolite of irinotecan
TAM: tumor-associated macrophages
TME: tumor microenvironment
TSC: tumor slice cultures
