# Supplementary material for: Tumor-associated macrophages and individual chemo-susceptibility are influenced by iron chelation in human slice cultures of gastric cancer
Source: Oncotarget. 2019 Jul 30;10(46):4731–42. doi: 10.18632/oncotarget.27089 (PMC6677664; doi:10.18632/oncotarget.27089)
Supplement: Supplementary file 1 [file oncotarget-10-4731-s001.pdf]

Tumor-associated macrophages and individual chemo-susceptibility are influenced by iron chelation in human slice cultures of gastric cancer

SUPPLEMENTARY MATERIALS

Supplementary Table 1: Clinical patient characteristics

Gender	Age, y	Sample ID	Tumor Location	Histolog. Type (Lauren)	Grading	TNM	Neoadjuvant Therapy	Response
Female	64	#2	Gastric Cancer, Antrum	Intestinal Type	G3	T1N0M0	ECX	minimal response
Female	67	#6	Gastric Cancer, Corpus	Intestinal Type	G2	T3N2M1	FLO + trastuzumab	minimal response
Female	65	#7	Gastric Cancer, Antrum	Diffuse Type	G3	T3N2M0	FLOT	minimal response
Male	56	#10	Gastric Cancer, Corpus	Diffuse Type	G3	T2N0M0	cisplatin *	not rated
Male	71	#12	Gastric Cancer, Antrum	Mixed Type	G2	T1N3M1	FLOT, FOLFIRI	minimal response
Male	68	#14	Gastric Cancer, Corpus	Diffuse Type	G3	T2N1M0	not treated	not treated
Male	77	#16	AEG Type II, Cardia	Diffuse Type	G2	T2N0M0	not treated	not treated

*RCT of oropharyngeal cancer. Minimal response to neoadjuvant treatment means from pathologists rated no significant tumor reduction or minimal response (>50% vital tumor cells; Werner and Höfler 2000).

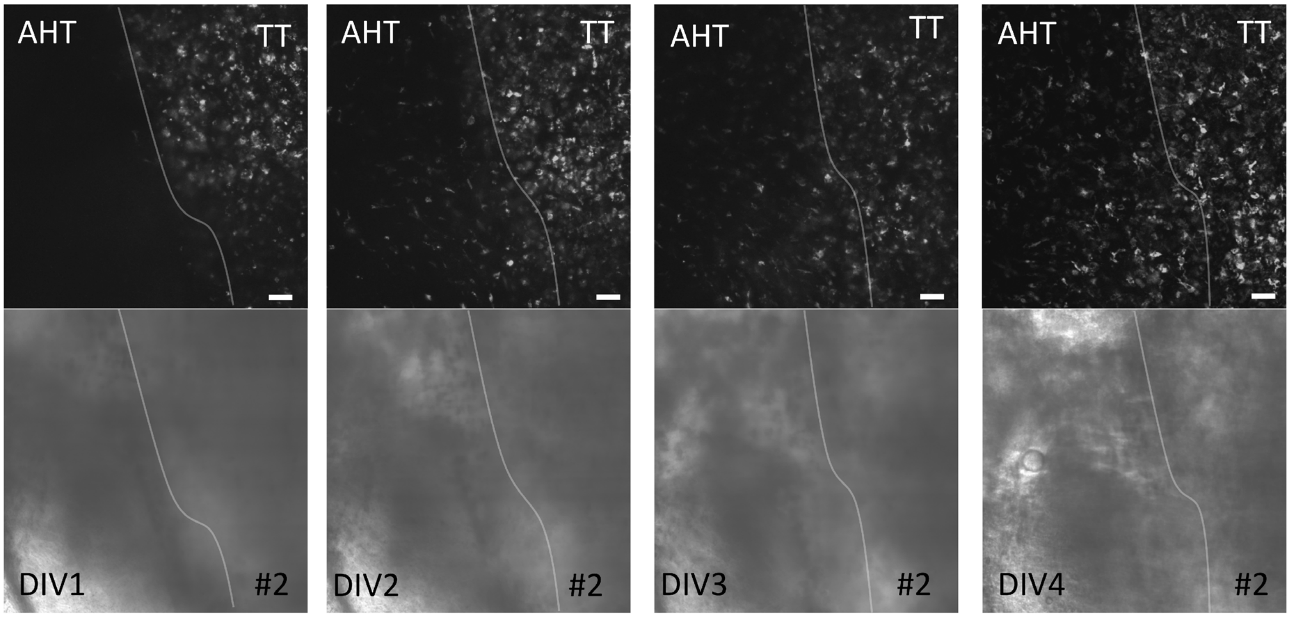

Supplementary Figure 1: TAM migration in co-cultures of human gastric tissue TAM (IB4, upper picture, gray scale) in tumor tissue (TT) migrate into adjacent healthy tissue (AHT) from specimen #2 after DIV1-4. The respective light microscopy picture is shown on the bottom of the fluorescent picture. (Bars 50 µm).

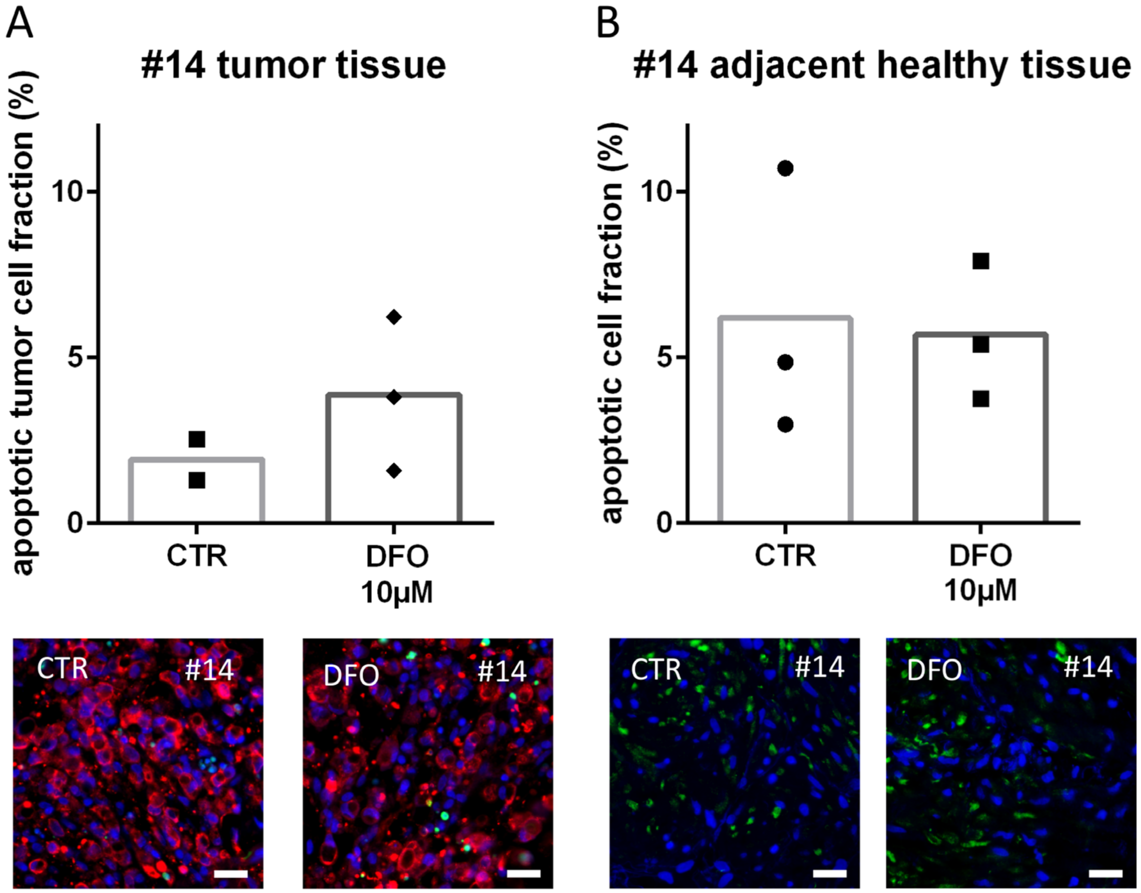

Supplementary Figure 2: Tumor tissue vs. adjacent healthy tissue after DFO 10 µM treatment. Apoptotic tumor cell fraction after 72h DFO (10 µM) treatment in gastric cancer slice cultures (**A**) and apoptotic cell fraction after 72 h DFO (10 µM) treatment in gastric adjacent healthy slice cultures (**B**) of specimen #14. Representative images of apoptotic tumor cell fraction in tumor tissue and adjacent healthy tissue are shown in the bottom of the graphs (nuclei, Hoechst; AE 1/3, red; cPARP, green). (Bars 20 µm).

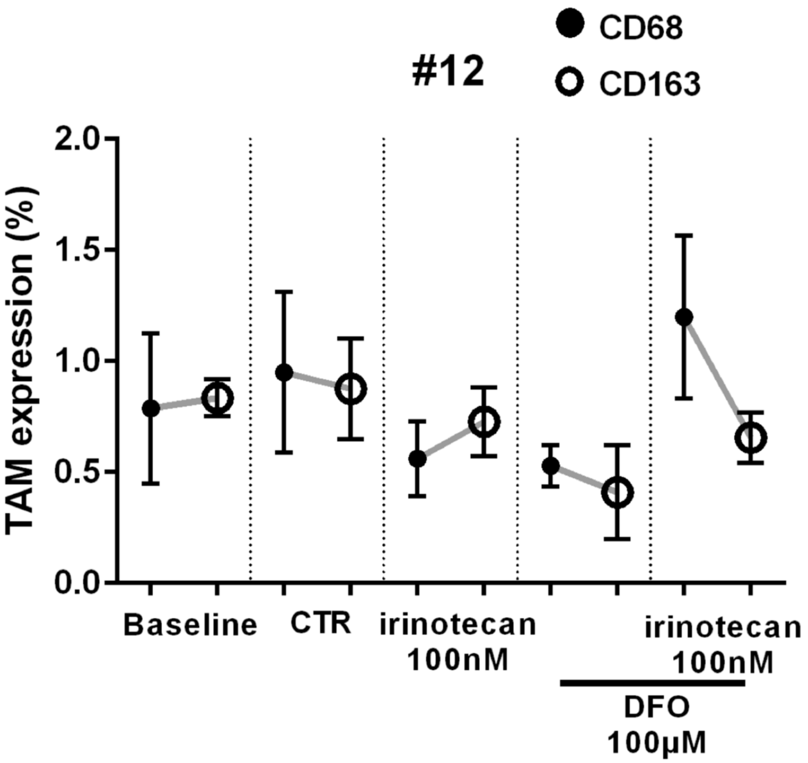

Supplementary Figure 3: TAM-phenotype expression after irinotecan and DFO treatment TAM (CD68; CD163) expression in overall tumor tissue after treatment in neoadjuvant treated specimen #12.

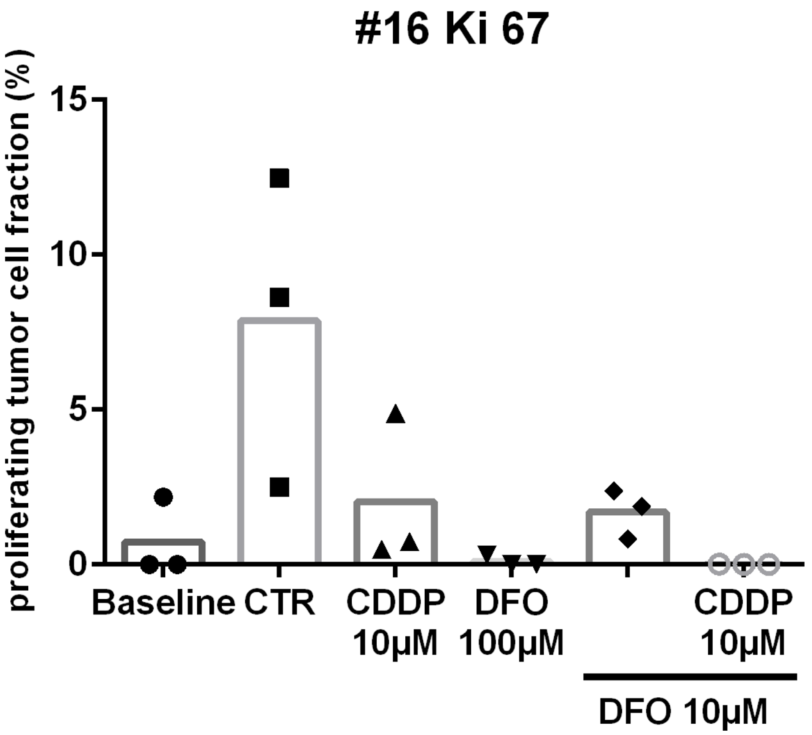

Supplementary Figure 4: Proliferating tumor cell fraction in specimen #16 Proliferating tumor cell fraction after treatment and DIV4 in chemo-naive specimen #16.

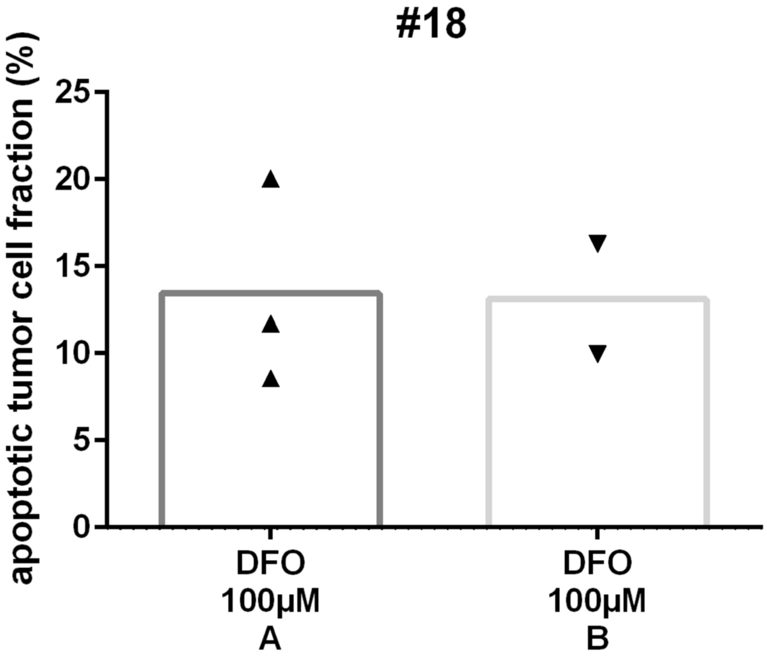

Supplementary Figure 5: Apoptotic tumor cell fraction in replicates of gastric tumor slice cultures Apoptotic tumor cell fraction after 72 h DFO (100 µM) treatment in tumor slice culture replicates.
